# The relationship between neuropsychiatric dimensions and markers of Parkinson’s disease risk in the UK Biobank

**DOI:** 10.1038/s41531-025-01181-y

**Published:** 2025-12-01

**Authors:** Bahaaeddin Attaallah, Sheena Waters, Charles Marshall, Alastair Noyce

**Affiliations:** 1 Centre for Preventive Neurology, Queen Mary University of London, London, UK; 2 Department of Brain Sciences, Imperial College London, London, UK

**Keywords:** Biomarkers, Diseases, Neurology, Neuroscience

## Abstract

Neuropsychiatric symptoms are a significant yet often overlooked aspect of Parkinson’s disease (PD). Using UK Biobank data, we examined associations between neuropsychiatric dimensions and PD risk markers. Factor analysis identified four dimensions—Depression, Anxiety, Adult Stress-Adversity, and Alcohol- and Substance-Related Behaviours (ASRB) —across three groups: PD, healthy controls, and cerebrovascular disease (CVD) as neurological controls. These dimensions showed distinct patterns in PD. Depression scores were significantly elevated, while ASRB scores were consistently lower. Neuroimaging linked ASRB to subcortical changes specific to PD, particularly quantitative susceptibility mapping in the substantia nigra, consistent with the dopaminergic system’s role in goal-directed behaviour. *GBA1* carrier status was linked to age-related changes in this dimension. Furthermore, PD patients with higher ASRB showed greater volatility in cognitive and motor function, with worsening before diagnosis and subsequent improvement. These findings highlight the complex interplay between psychiatric symptoms, neurobiological changes, and genetic factors in PD, suggesting that specific neuropsychiatric profiles may serve as early indicators of disease risk and progression.

## Introduction

Parkinson’s disease (PD) is a progressive neurodegenerative disorder characterised by motor symptoms such as tremor, rigidity, and bradykinesia, as well as a wide range of non-motor symptoms^1–3^. Among these non-motor manifestations, neuropsychiatric symptoms are increasingly recognised as important features of PD, often preceding the onset of motor symptoms by years or even decades^4–6^.

The complex relationship between neuropsychiatric symptoms and PD has been a subject of growing interest in recent years. Depression, anxiety, and apathy are commonly observed in PD patients, with prevalence rates significantly higher than in the general population^7,8^. Epidemiological studies have suggested that a history of depression or anxiety may be associated with an increased risk of developing PD later in life^9–11^. Depression in PD has also been linked to increased dementia risk, higher mortality, and reduced subcortical brain volumes^12^. However, the precise nature of this relationship remains to be fully understood, particularly when considering the interplay of pathological changes and genetic predispositions. Additionally, the potential role of other neuropsychiatric dimensions, such as addiction and stress response, warrants further investigation^5,8^.

Recent advances in neuroimaging and genetic research have provided new opportunities to explore the neurobiological underpinnings of both PD and neuropsychiatric disorders. Structural and functional brain changes associated with PD have been extensively documented, including alterations in subcortical regions such as the substantia nigra, striatum, and various limbic structures^13–15^. These regions are differentially implicated in affect regulation, motivation, reward processing, and goal-directed behaviour—functions often disrupted in both PD and psychiatric conditions^5,16–19^. Similarly, neuroimaging studies of psychiatric disorders have revealed abnormalities in overlapping brain circuits, suggesting potential shared neurobiological mechanisms^20,21^. These observations underscore the importance of delineating distinct neuropsychiatric dimensions and linking them to specific neural substrates in order to better understand the heterogeneity of psychiatric symptoms in PD and their relevance to disease pathophysiology.

Genetic factors also play a crucial role in both PD and mental health. Mutations in genes such as *SNCA*, *LRRK2*, and *GBA1* have been implicated in PD risk and differential PD progression^22^. Interestingly, some of these genetic variants have also been associated with psychiatric symptoms, raising questions about potential shared genetic determinants of both PD and neuropsychiatric disorders^23,24^. By contrast, the APOE ε4 allele is best known as the major genetic risk factor for Alzheimer’s disease but has also been implicated as a modifier of cognitive decline and dementia risk in PD in multiple studies^25–27^. Examining APOE ε4 alongside PD-specific genes such as GBA1 therefore, helps to determine whether observed associations with neuropsychiatric dimensions reflect mechanisms specific to PD-linked genetics (GBA1) or are driven by genetic susceptibility to dementia more generally (APOE ε4); several longitudinal and cohort studies report independent and sometimes additive effects of GBA1 and APOE ε4 on cognitive outcomes, supporting this comparative approach^28^.

The UK Biobank is a powerful resource for investigating the complex relationships between mental health, neurobiological markers, and PD. This large-scale prospective cohort study provides a wealth of data on health-related outcomes, including detailed mental health assessments, neuroimaging measures, and genetic information.

In this study, we used UK Biobank data to clarify how neuropsychiatric features relate to clinical, imaging and genetic markers of PD risk. Specifically, we sought to I) identify distinct neuropsychiatric dimensions through factor analysis of comprehensive mental health questionnaires; II) examine whether the trajectories of these neuropsychiatric dimensions differ between people with PD, healthy controls (HCs), and a neurological control group (cerebrovascular disease; CVD); III) investigate which neuroimaging signatures in subcortical structures implicated in PD (for example, susceptibility and microstructural changes in the substantia nigra and basal ganglia) map onto specific neuropsychiatric dimensions; IV) determine whether genetic risk variants for PD (notably loci such as GBA1) relate to these neuropsychiatric dimensions. By integrating these diverse data streams, we aim to determine whether particular neuropsychiatric profiles may serve as early indicators of PD risk and to disentangle mechanisms that are unique to PD from those that are shared across neurological diseases.

## Results

### Participants

A total of 93,898 HC participants had sufficient data available to perform factor analysis (Age: *µ* = 63.8*, SD* = 7.65). Among the participants diagnosed with PD (*N* = 4571), factor scores were successfully derived for 696 individuals (Age: *µ* = 69.45*, SD* = 5.5). Similarly, from an initial group of 36,875 participants diagnosed CVD, factor scores were available for 6560 individuals (Age: *µ* = 68.47*, SD* = 6.55). Table 1 shows demographics of the study groups.

**Table 1:** Study demographics

	HC	CVD	PD
Sample size	93,898	6560	696
Gender (F)	52,000 (55.4%)	2821 (43.0%)	267 (38.4%)
Age (mean *±* SD)	63.87 ± 7.65	68.47 ± 6.55	69.45 ± 5.50
Education (mean *±* SD)	7.18 ± 3.00	6.80 ± 3.41	7.19 ± 3.41
Ethnicity			
White	90,965 (96.9%)	6396 (97.5%)	684 (98.3%)
Non-white	2649 (2.8%)	142 (2.2%)	9 (1.3%)
Unknown	284 (0.3%)	22 (0.3%)	3 (0.4%)
MRI			
Data available	16934 (18.0%)	947 (14.4%)	64 (9.2%)
No data	76964 (82.0%)	5613 (85.6%)	632 (90.8%)
*GBA1*			
Carrier	4067 (4.3%)	290 (4.4%)	47 (6.8%)
Non-carrier	87430 (93.1%)	6117 (93.4%)	635 (91.2%)
Unknown	2401 (2.6%)	153 (2.3%)	14 (2.0%)
*APOE4*			
Carrier	1946 (2.1%)	155 (2.4%)	14 (2.0%)
Non-carrier	89821 (95.7%)	6273 (95.6%)	671 (96.4%)
Unknown	2131 (2.3%)	132 (2.0%)	11 (1.6%)

*HC* controls, *CVD* cerebrovascular disease. *PD* Parkinson’s disease. *F* female. Age and education are in years.

### Latent neuropsychiatric dimensions identified via factor analysis

**Fig. 1:**
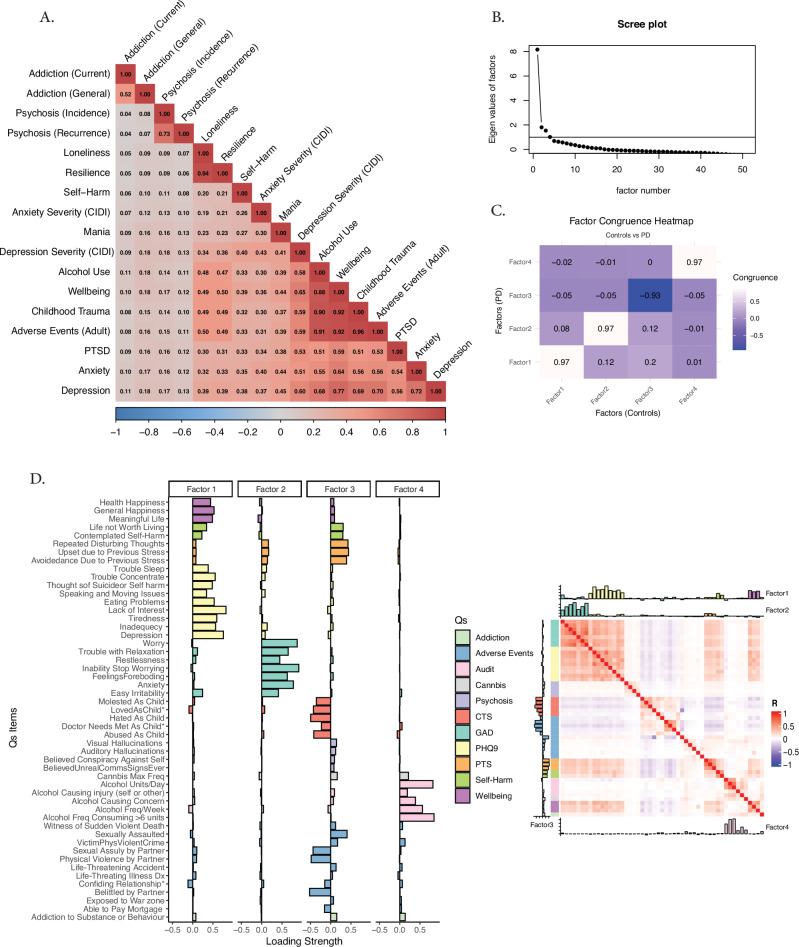
Factor analysis and neuropsychiatric dimensions. **A** Significant correlation among mental health questionnaire scores in the UK Biobank cohort. **B** Scree plot indicating support for a four-factor solution derived from 51 items across 11 questionnaires. **C** Congruence map comparing factor structures between control participants and individuals with Parkinson’s disease (PD), demonstrating high consistency across groups, with absolute coefficient values exceeding 0.9. **D** Item loadings onto the four identified factors: I) Depression, II) Anxiety, III) Adult Stress-Adversity, and IV) Alcohol- and Substance-Related Behaviours (ASRB). The panel on the right depicts the inter-item correlation matrix. CTS childhood trauma questionnaire, GAD generalised anxiety disorder questionnaire, PHQ-9 patient health questionnaire-9, PTSD post-traumatic stress disorder. To ensure consistent interpretations across groups, Factor 3 scores for PD were reversed in subsequent analyses and visualisations.

Seventeen neuropsychiatric measures were extracted from the two main mental health questionnaires (MHQ1 and MHQ2) within the UK Biobank dataset. These measures displayed substantial intercorrelations, complicating efforts to evaluate and interpret their individual contributions independently (Fig. 1A).

To address multicollinearity and uncover underlying constructs, exploratory factor analysis was conducted. For statistical robustness and improved stability, the analysis included 51 eligible items aggregated from 11 (out of the original 17) mental health-related questionnaires. Inspection of the scree plot (Fig. 1B) and eigenvalue criteria supported a four-factor solution. The resulting factors were interpreted as: (I) Depression, (II) Anxiety, (III) Adult Stress Adversity, and (IV) Alcohol- and Substance-Related Behaviours (ASRB) (Fig. 1D). The Depression dimension primarily encompassed all items from the PHQ-9 questionnaire as well as items related to subjective well-being. The Anxiety dimension was predominantly defined by items from the GAD questionnaire. The Stress-Adversity factor consisted of items derived from the Childhood Trauma Screener (CTS), PTSD, and traumatic life event questions. The fourth factor (ASRB) included items primarily from the AUDIT questionnaire and, to a lesser extent, questions related to cannabis use and addictive behaviours, though the latter exhibited weaker loadings.

The factor structure demonstrated high consistency across the control group and the two clinical populations (PD and CVD) with absolute congruence coefficients exceeding 0.90 (Fig. 1C), indicating comparable underlying mental health dimensionality across these populations.

### Trajectories of neuropsychiatric dimensions in PD

**Fig. 2:**
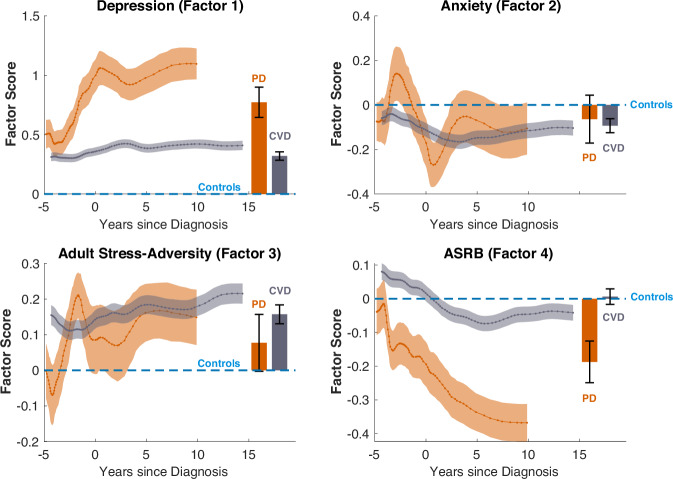
Trajectories of neuropsychiatric dimensions in Parkinson’s disease (PD). Conditional plots depicting age-residualised factor scores for PD and cardiovascular disease (CVD), alongside bar plots illustrating group mean scores with 95% confidence intervals (CIs). Both PD and CVD groups exhibit significantly higher depression scores relative to controls (dashed horizontal line at zero). While PD and CVD show comparable depression levels several years prior to diagnosis, depression scores in PD increase markedly pre-diagnosis, resulting in overall significantly higher levels compared to CVD, in whom scores remain relatively stable. CVD participants exhibit lower anxiety and higher adult stress-adversity scores compared to controls, but not significantly different from PD. Additionally, PD patients show significantly lower ASRB scores relative to both CVD and control groups, with scores continuing to decline as the disease progresses. Shaded areas around the trajectories represent 95% CIs. ASRB alcohol- and substance-related behaviours.

We next examined the trajectories of the identified neuropsychiatric dimensions in individuals with PD, using conditional plots to compare factor scores across the PD, CVD, and HC groups. Depression dimension scores were significantly elevated in the PD group relative to both CVD and control groups (HC vs PD: *β* = *−*0.804, *SE* = 0.0443, *t*_99530_ = *−*18.14, *p* < 0.0001; CVD vs PD: *β* = *−*0.453, *SE* = 0.0464, *t*_99530_ = *−*9.77, *p* < 0.0001; Table S5), whereas ASRB scores were consistently lower in PD (HC vs PD: *β* = +0.271, *SE* = 0.0331, *t*_99530_ = +8.19, *p* < 0.0001; CVD vs PD: *β* = +0.218, *SE* = 0.0346, *t*_99530_ = +6.30, *p* < 0.0001; Fig. 2).

These differences were evident several years prior to PD diagnosis and persisted throughout the disease course; depression scores continued to rise post-diagnosis, while ASRB scores declined further during later stages.

By contrast, individuals with CVD exhibited relatively stable depression scores, which were elevated compared to controls but consistently lower than those observed in PD. Similarly, their ASRB scores remained comparable to controls throughout the time course.

Regarding anxiety (Factor 2) and adult stress-adversity (Factor 3), no significant differences in anxiety scores were observed between PD and control groups (HC vs PD: *β* = *−*0.0341, *SE* = 0.0464, *t*_99530_ = *−*0.73, *p* = 0.46), nor between PD and CVD. However, stress adversity scores were significantly higher in PD compared to controls (HC vs PD: *β* = +0.222, *SE* = 0.0365, *t*_99530_ = +6.09, *p* < 0.0001) but did not significantly differ from CVD. Both dimensions exhibited modest increases in the years preceding PD diagnosis, followed by gradual declines thereafter.

### Subcortical neuroimaging correlates of neuropsychiatric dimensions

To elucidate the neurobiological substrates of distinct neuropsychiatric dimensions, we investigated associations between subcortical volumes, T2* signal, and quantitative susceptibility mapping (QSM) measures with the four identified neuropsychiatric factors across the groups.

**Fig. 3:**
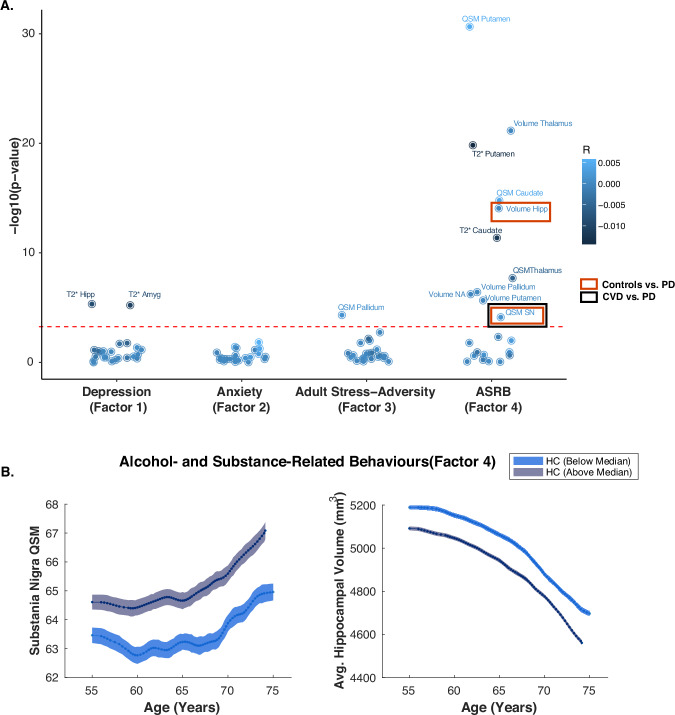
Associations between subcortical neuroimaging measures and neuropsychiatric factor scores. **A** Partial correlations between subcortical indices (volume, QSM, T2*) and neuropsychiatric factor scores. Notably, the depression dimension was negatively correlated with T2* signal in limbic regions, specifically the hippocampus and amygdala. The ASRB dimension exhibited significant associations with multiple subcortical measures, including increased QSM values in the substantia nigra (SN) and reduced hippocampal volume. Additionally, SN QSM values were significantly elevated in PD patients relative to both the control and CVD groups. No significant correlations were observed for the anxiety dimension after Bonferroni correction, while the adult stress-adversity dimension showed a significant association with QSM values in the pallidum. **B** Conditional plots depicting SN QSM values (left) and hippocampal volumes (right) as a function of age. Trajectories are stratified by higher versus lower ASRB scores (median split), illustrating consistently higher SN QSM values and lower hippocampal volumes in individuals with higher dimension scores across the age range. All correlations were corrected for multiple comparisons using the Bonferroni method (92 comparisons) and adjusted for age, gender, education, assessment centre, and the time difference between MRI acquisition and questionnaire completion. Data shown for the HC group. For full statistical details across groups, see Table S7. Amyg amygdala, ASRB alcohol- and substance-related behaviours, Hipp hippocampus. SN, substania nigra, QSM quantitative susceptibility mapping.

A prominent pattern of significant associations emerged between the ASRB dimension and various subcortical indices. Elevated scores on this dimension were strongly associated with reduced bilateral average subcortical volumes (Fig. 3A, Table S7), specifically in the thalamus (*β* = *−*0.000086, adjusted *p* < 0.0001), hippocampus (*β* = *−*0.000089, adjusted *p* < 0.0001), pallidum (*β* = *−*0.000126, adjusted *p* < 0.0001), putamen (*β* = *−*0.000054, adjusted *p* < 0.0001), and nucleus accumbens (*β* = *−*0.000251, adjusted *p* < 0.001).

Additionally, QSM analyses revealed significant alterations in magnetic susceptibility, suggestive of iron accumulation or changes in tissue composition, in individuals with higher ASRB scores. Specifically, increased QSM values were observed in the putamen (*β* = 0.0049, adjusted *p* < 0.0001), substantia nigra (*β* = 0.001647, adjusted *p* = 0.0052), and thalamus (*β* = *−*0.0051, adjusted *p* < 0.0001). Notably, while increased susceptibility typically indicates greater iron deposition, the negative QSM correlation in the thalamus may reflect more complex microstructural or mineralisation changes, potentially linked to dysregulated dopaminergic pathways implicated in addiction.

Complementing these findings, higher ASRB scores were also associated with reduced T2* signal in the putamen (*β* = *−*0.013, adjusted *p* < 0.0001) and caudate (*β* = *−*0.011, adjusted *p* < 0.0001). Reduced T2* relaxation times are generally consistent with elevated iron concentrations or altered tissue microstructure, supporting the QSM findings and suggesting converging evidence of subcortical iron dysregulation in addiction-related phenotypes.

Regarding the depression dimension, significant negative associations were identified between depressive symptoms and T2* signal in key limbic regions. Specifically, higher depression scores correlated with reduced T2* signal in the hippocampus (*β* = *−*0.01, adjusted *p* < 0.001) and amygdala (*β* = *−*0.0083, adjusted *p* < 0.001), suggestive of increased iron accumulation or altered microstructure in these emotion-regulation regions.

The adult stress-adversity dimension showed positive, though non-significant after correction, associations with QSM values in several subcortical regions, including substantia nigra and pallidum. While these associations did not survive multiple comparisons correction, the pattern may indicate stress-related alterations in iron metabolism or subcortical microstructure, warranting further investigation.

No significant correlations were identified between the anxiety dimension and any subcortical neuroimaging measures after adjusting for multiple comparisons (all adjusted *p* > 0.99).

This suggests that, within the current dataset and methodological framework, anxiety symptoms may not exhibit strong associations with subcortical structural or iron-related changes.

Finally, group comparisons revealed significant subcortical neuroimaging differences associated with PD. Specifically, individuals with PD exhibited significantly higher QSM values in the substantia nigra compared to both control participants (*β* = *−*8.55*, SE* = 1.99*, t*_17910_ = −4.29*, p* < 0.0001) and individuals with CVD (*β* = *−*6.90*, SE* = 2.05*, t*_17910_ = *−*3.36*, p* = 0.00077). This result aligns with the well-established observation of increased iron accumulation in the substantia nigra in PD, a neuropathological hallmark implicated in oxidative stress and dopaminergic neuronal degeneration^29,30^.

Additionally, hippocampal volume was significantly reduced in the PD group relative to controls (*β* = +206*, SE* = 61.1*, t*_19951_ = +3.37*, p* = 0.00076), suggesting possible hippocampal involvement in PD-related neurodegeneration, consistent with previous findings highlighting cognitive and memory deficits in PD^31,32^. No significant differences in hippocampal volume were observed between PD and CVD groups (*p* = 0.11), and no other subcortical indices showed significant group differences after correction for multiple comparisons (*p* > 0.05).

### Genetic associations with neuropsychiatric dimensions

The association between genetic status (*GBA1* and APOE ε4 carrier status) and the four Neuropsychiatric dimensions (depression, anxiety, adult stress-adversity, ASRB) was assessed using mixed-effects models. Each model included age, gender, education, and interaction terms between genetic status and age to explore potential age-dependent effects.

**Fig. 4:**
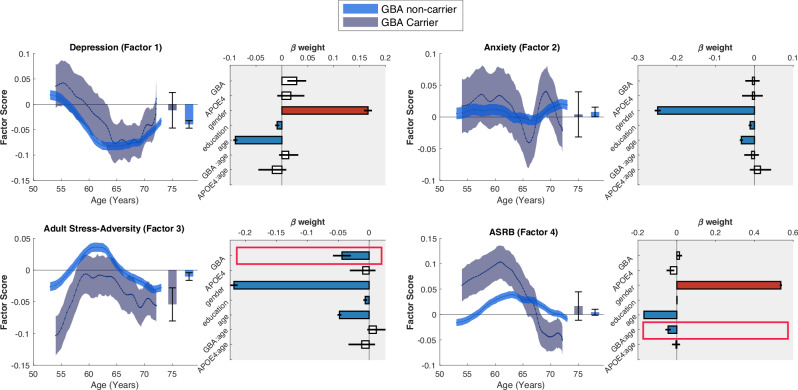
Effect of *GBA1* carrier status on neuropsychiatric dimensions. No significant differences were observed between *GBA1* carriers and non-carriers for the depression (Factor 1) and anxiety (Factor 2) dimensions (both *p* > 0.05). *GBA1* carriers exhibited significantly lower adult stress-adversity scores (Factor 3) compared to non-carriers (main effect: *β* = *−*0.0404, *SE* = 0.0141, *t*_98360_ = *−*2.86, *p* = 0.0042; bottom left panel). Additionally, a significant interaction between *GBA1* carrier status and age was identified for the ASRB dimension (Factor 4), whereby *GBA1* carriers showed higher scores in early adulthood (50 s) that declined markedly with age, relative to a stable trajectory in non-carriers (*GBA1* × age interaction: *β* = *−*0.0404, *SE* = 0.0136, *t*_98360_ = *−*2.96, *p* = 0.0031; bottom right panel). Notably, no significant main or interaction effects of APOE ε4 carrier status were observed on any of the dimensions (all *p* > 0.05). Data shown represent the control group. For full statistical details and model specifications across all groups, see Table S9. ASRB alcohol- and substance-related behaviours.

A significant main effect of *GBA1* carrier status was observed for the adult stress-adversity dimension (Factor 3), with *GBA1* carriers exhibiting lower stress-adversity scores compared to non-carriers (*β* = *−*0.0404, *SE* = 0.0141, *t*_98360_ = *−*2.86, *p* = 0.0042, Fig. 4, Table S9). Furthermore, a significant interaction between *GBA1* carrier status and age was found for the ASRB dimension (Factor 4) (*β* = *−*0.0404, *SE* = 0.0136, *t*_98360_ = *−*2.96, *p* = 0.0031).

Specifically, *GBA1* carriers displayed higher ASRB scores in early adulthood (i.e., in their 50 s), which declined significantly with advancing age, whereas non-carriers showed a more stable trajectory. This age-dependent pattern may provide a potential explanation for the lower ASRB scores observed in older individuals with PD, who concurrently demonstrated higher substantia nigra QSM values (Fig. 3A).

No significant main or interaction effects of APOE ε4 carrier status were detected on any of the neuropsychiatric dimensions (all *p* > 0.05), suggesting that the genetic associations observed are specific to *GBA1* carrier status rather than generalisable to other common genetic risk variants.

These findings underscore the potential role of GBA1 genetic variation in shaping age-related trajectories of stress-adversity and addiction-related traits, possibly reflecting prodromal neuropsychiatric profiles associated with PD risk.

### Influence of ASRB on clinical attributes of Parkinsonism

Given previous analyses showing an association between PD neuroimaging and genetic markers with Factor 4 scores, we next examined how this dimension interacts with key clinical attributes potentially affected in Parkinsonism. This included executive function, assessed via confirmatory factor analysis (CFA) of four tasks: snap reaction time, trail-making task, digit symbol substitution test, and tower rearranging task, alongside handgrip strength and motor symptoms (bradykinesia, rigidity, tremor, falls, gait, and balance impairments).

**Fig. 5:**
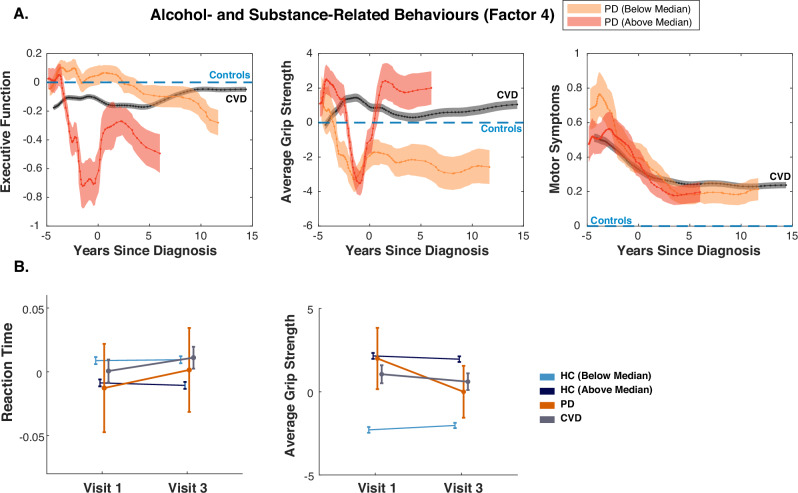
Effect of factor 4 (ASRB) on symptoms associated with PD. **A**. PD patients with higher ASRB scores show a decline in executive function before diagnosis, which then improves soon after diagnosis. A similar pattern is observed for grip strength. This may suggest a higher sensitivity in these PD patients to disease processes and interventions. Motor symptom reports did not change with these dimension scores, which may be due to the poor quality and specificity of this measure in capturing PD symptoms within the dataset. **B**. Longitudinally, both reaction time and grip strength worsened across groups. ASRB scores did not affect reaction time between visits, while they had a significant effect on grip strength, with higher scores associated with a faster rate of decline. This faster rate of decline is also observed in PD patients compared to the other groups.

Executive function declined with age across all groups (*Age*: *β* = *−*0.385*, SE* = 0.00475*, t*_22415_ = *−*81.01*, p* < 0.0001, Fig. 5A, Table S10). However, in PD patients, higher ASRB scores prior to diagnosis were associated with an accelerated age-related decline in executive function (*Factor*4 *×* *Age* (*pre-diagnosis*): *β* = 0.326*, SE* = 0.136*, t*_42_ = 2.40*, p* = 0.021, Table S11). Interestingly, after diagnosis, this trend reversed, with some improvement observed (*Factor*4 *×* *Age* (*post-diagnosis*): *β* = *−*0.374*, SE* = 0.211*, t*_23_ = *−*1.78*, p* = 0.09), although this did not reach significance. This improvement may suggest a treatment effect or increased responsiveness to interventions post-diagnosis. Surprisingly, in HCs, higher ASRB scores were associated with better executive function (*HC*: *β* = 0.0347*, SE* = 0.00505*, t*_21243_ = 6.70*, p* < 0.0001), with no significant interaction with age (*HC*; *Factor*4 *×* *Age* : *β* = 0.0072*, SE* = 0.0049*, t*_21243_ = 1.42*, p* = 0.15). While reaction time (the only measure from executive function derived score captured at baseline) also declined longitudinally between visits (*β* = 0.11*, SE* = 0.001*, t*_58317_ = 8.39*, p* < 0.0001, Fig. 5B, Table S12), the decline did not seem to be affected by ASRB in PD (*PD* *×* *visit*: *p* = 0.16). Suggesting that the effect of ASRB on executive function is related to other measures the score captures, which was indeed the case, mainly the trail-making task (*PD*. *Factor*4*effect* : *β* = −0.044*, SE*=0.016*, t*_69_ = *−*2.7*, p* = 0.006).

Grip strength declined with age across participants (*Age*: *β* = *−*2.15*, SE* = 0.0366*, t*_29070_ = −58.75*, p* < 0.0001). In PD patients, grip strength appeared to dip just before diagnosis, regardless of factor 4 score, but remained stable in CVD patients. Notably, PD patients with higher ASRB scores showed significant improvement in grip strength with age compared to those with lower scores (*Factor*4 *×* *Age* (*post−diagnosis*): *β* = *−*2.4*, SE* = 1*, t*_42_ = −2.40*, p* = 0.021). This suggests higher sensitivity to treatment in patients with higher ASRB scores. HCs with higher factor scores showed a stronger grip compared to those with lower scores. However, longitudinally, grip strength declined at a faster rate in HCs with higher scores (*Visit* *×* *HC*(*belowFactor*4*median*):*β* = 0.469*, SE* = 0.079*, t*_59047_ = 5.9*, p* < 0.0001), and this rate of decline was significantly higher in PD patients (*Visit×PD*: *β* = *−*2.17*, SE* = 0.623*, t*_59047_ = *−*3.48*, p* < 0.001).

There was no significant main effect of the ASRB dimension or interaction with age on motor symptoms. This might be due to the poor temporal correlation between this measure and factor scores, as it captures whether participants ever reported such symptoms. Additionally, it does not differentiate between different types of tremor or balance issues.

Overall, these findings indicate that the impact of ASRB on clinical measures in PD patients is dynamic, with potential worsening before diagnosis but some improvements post-diagnosis. Conversely, in HCs, higher ASRB scores were associated with better executive function and grip strength.

## Discussion

This study provides novel insights into the relationship between neuropsychiatric dimensions and markers of PD risk, leveraging the extensive UK Biobank dataset. By identifying four latent neuropsychiatric dimensions: depression, anxiety, adult stress-adversity, and ASRB, and examining their trajectories, neuroimaging correlates, and genetic associations, we show how these dimensions evolve in the course of PD in comparison to healthy people and another disease group–CVD. The ASRB dimension showed consistent inverse associations across different aspects related to PD, including substantial nigra integrity, genetic risk, as well as clinical attributes such as executive function and grip strength. Higher depression scores in PD, observed both pre- and post-diagnosis, are consistent with previous research identifying depression as a common prodromal symptom of PD^1,9^. Depression not only precedes motor symptoms but also predicts poorer quality of life (QoL) and increased caregiver burden in PD patients^33–35^. Our findings corroborate these observations, demonstrating that depression trajectories diverge significantly between PD patients and other groups, with a marked increase as the disease progresses. This supports the hypothesis that depression is not merely reactive to a PD diagnosis but reflects underlying neurobiological changes, such as dopaminergic and serotonergic dysfunction or, as reported in this study, limbic regions involvement^7,36^.

PD patients had higher adult stress-adversity (Factor 3) scores, compared to HCs, but not different from CVD. This latent construct is characterised by increased reports of current PTSD symptoms and self-harm alongside reduced endorsement of items from the short CTS. The PCL-S assesses current post-traumatic stress symptomatology rather than the timing of antecedent events, whereas the CTS is an abbreviated retrospective screener derived from the Childhood Trauma Questionnaire and therefore does not capture detailed information on timing, chronicity or context of maltreatment PTSD^37^. Several non-mutually exclusive explanations may account for the observed increase in this factor observed to start around the time of diagnosis. First, elevated PCL-S scores may reflect adult traumatic exposures or reactivation of trauma-related symptoms (note that a PD diagnosis can be a traumatic experience) in adulthood rather than new childhood events. Second, retrospective reports of childhood maltreatment frequently disagree with prospectively obtained records and are susceptible to mood and symptom-congruent recall or response biases; thus, lower CTS endorsement contemporaneous with increased current symptoms may reflect reporting processes rather than an absence of earlier adversity^38^. Third, PTSD commonly co-occurs with depressive disorders and is associated with increased risk of self-harm and suicidal behaviour, so the factor may partly index broader internalising/affective distress that becomes more salient near diagnostic assessment^39^. Finally, the ultra-short format of the CTS necessarily limits the granularity with which trauma type and timing are captured. For these reasons, we urge cautious interpretation: higher “adult stress-adversity” scores should be read as reflecting greater current PTSD symptoms and self-harm with reduced endorsement on brief childhood-trauma items, a profile plausibly arising from adult trauma exposure, psychopathology-related reporting effects, or both. Future studies using prospective trauma ascertainment or comprehensive lifetime event inventories would help disentangle timing from reporting bias.

ASRB (Factor 4) scores were consistently lower in PD patients compared to controls and CVD. Several epidemiological studies have reported an inverse association between alcohol consumption and PD risk, suggesting that individuals who consume moderate to high amounts of alcohol may have a lower likelihood of developing the disease^40–42^. However, findings are inconsistent, with some large prospective studies observing no significant association between alcohol intake and PD risk^43^. These discrepancies may stem from methodological differences, including study design, population characteristics, and potential confounding factors.

Despite the apparent paradox between lower ASRB (Factor 4) scores in the PD group and the well-recognised occurrence of impulsive-compulsive behaviours in PD, several methodological and aetiological factors plausibly explain this finding. First, ICDs in PD are strongly associated with dopaminergic treatment, particularly dopamine agonists, rather than the diagnosis per se, so measured ICD prevalence will depend heavily on medication exposure^44,45^. Second, this factor may be driven predominantly by alcohol-use items in the questionnaire and therefore fail to capture medication-related behavioural ICDs for which PD-specific instruments such as the QUIP and QUIP-RS are more sensitive^46–48^. Third, self-completed instruments are prone to under-reporting of socially sensitive behaviours and can diverge from caregiver or clinician assessments, biasing prevalence estimates downward when only patient self-report is available^49,50^. Finally, alcohol consumption commonly falls around the time of diagnosis, which may itself produce lower measured alcohol-related scores among diagnosed patients compared with community controls^51^. These considerations should be accounted for when interpreting results for this factor, and caution should be exercised before generalising them to broader goal-directed behaviour deficits in PD.

The observed lower ASRB scores among PD patients may reflect preclinical behavioural and motivational changes rather than a direct protective effect of alcohol. PD is characterised by alterations in reward processing and goal-directed behaviour, potentially leading to a decreased inclination toward reward-seeking and addictive behaviours as the disease progresses^52,53^. This interpretation aligns with evidence of motivational deficits, such as apathy, which are common in PD^54,55^. At the same time, it may also indicate an underlying vulnerability to impulsive and addictive behaviours, consistent with emerging views that apathy and impulsivity represent two sides of the same coin—both stemming from dysregulation within overlapping cortico-striatal circuits affected by neurodegeneration^56–58^. From this perspective, the ASRB dimension is likely to be inherently volatile, shaped by bidirectional shifts in motivational control. Considering the dynamic changes in these behavioural traits with age and neurodegenerative progression, particularly in *GBA1* carriers, the observed pattern may reflect broader volatility in goal-directed behaviour.

These behavioural and motivational changes could also explain the more pronounced fluctuations in executive function scores and handgrip strength before and after diagnosis. As PD affects fronto-striatal circuits, alterations in executive function and motor control may emerge early in the disease course and become more pronounced over time^59^. Importantly, these behavioural shifts may be particularly sensitive to both disease pathology and subsequent interventions (Fig. 5), highlighting the need for further longitudinal studies to disentangle the complex interactions between addiction-related behaviours, neurodegeneration, and compensatory mechanisms in PD.

Our neuroimaging analyses revealed significant associations between neuropsychiatric dimensions and subcortical brain measures. Depression was linked to reduced T2* signal in limbic regions such as the hippocampus and amygdala, indicative of iron accumulation or altered microstructure. These findings align with prior studies implicating hippocampal atrophy and iron dysregulation in mood disorders^15,60,61^. Similarly, higher ASRB scores were associated with volumetric reductions in subcortical structures like the thalamus and hippocampus, as well as increased magnetic susceptibility in the substantia nigra. These patterns suggest shared neurobiological mechanisms underpinning addiction-related behaviours and PD pathology, possibly mediated by dopaminergic dysfunction or oxidative stress^30^.

Our genetic analyses identified significant interactions between *GBA1* carrier status and age-related changes in ASRB dimension. *GBA1* mutations are well-established risk factors for PD and are associated with earlier onset and more severe non-motor symptoms^62^. The observed decline in ASRB scores among older *GBA1* carriers suggests potential age-dependent shifts in reward sensitivity or behavioural regulation, similar to what is seen in PD patients. In contrast, no significant associations were observed for APOE ε4, a genetic risk factor for Alzheimer’s disease^63^. This specificity underscores the distinct genetic underpinnings of neuropsychiatric trajectories in different neurodegenerative contexts.

The results of this study are unlikely to be significantly influenced by the COVID-19 pandemic, as the factor analysis primarily utilised items from the Mental Health Questionnaire (MHQ1), which was administered prior to the pandemic in 2016. While the Mental Well-being Follow-up Questionnaire (MHQ2), collected post-pandemic in 2022, provided additional data, many items were excluded due to redundancy, low variance and sample size, or other methodological considerations. This exclusion minimises direct pandemic-related effects on the identified neuropsychiatric dimensions. Nevertheless, it is important to consider the broader implications of COVID-19 on mental health outcomes in PD. Studies have shown that PD patients experienced heightened psychological distress during the pandemic, with increased rates of depression and anxiety compared to controls, largely driven by social isolation, disrupted healthcare access, and reduced physical activity^64^. These stressors may exacerbate pre-existing vulnerabilities in PD patients and influence mental health trajectories indirectly. Although the methodological limitations of MHQ2 reduce direct pandemic-related confounding in this study, future research should explore how long-term effects of COVID-19, such as chronic stress and altered healthcare delivery, might shape neuropsychiatric outcomes in PD populations.

This study offers important insights into neuropsychiatric dimensions in a large, well-characterised population cohort. A major strength is the integration of mental health phenotyping with multimodal neuroimaging and genetic data in the UK Biobank, enabling the identification of transdiagnostic markers that are rarely accessible in clinical samples. The population-based design, coupled with ongoing longitudinal follow-up, supports the potential characterisation of these dimensions in prevalent and incident cases. However, several limitations warrant consideration.

Mental health data were obtained via self-report, which may introduce recall and reporting biases, particularly for stigmatised conditions such as addiction or psychosis. Moreover, while the UK Biobank is broadly representative in terms of geographic and demographic diversity, it exhibits a well-documented healthy volunteer bias: participants are more likely to be white, highly educated, and in better physical and mental health than the general population^65,66^. This may lead to underestimation of mental health burden and reduced generalisability to more clinically affected groups. Another consideration is the reliance on routinely collected primary care and hospital episode statistics, as well as self-reports, for diagnostic data. These records vary in completeness and may not capture undiagnosed or subclinical cases. In addition, diagnostic dates may reflect time of record entry rather than symptom onset, complicating temporal interpretations. Further longitudinal studies are crucial to validate the findings and replicate temporal relationships. Future research should also explore additional neuropsychiatric dimensions, such as motivational deficits, and their interplay with cognitive function and underlying brain networks. Expanding analyses to more diverse populations will enhance generalisability while addressing disparities in diagnostic access and treatment availability, ultimately improving the translational relevance of these findings.

In conclusion, this study highlights the distinct trajectories of neuropsychiatric dimensions in PD, with elevated depression and lower alcohol- and substance-related behaviours scores. The alcohol- and substance-related dimension was linked to PD-related subcortical brain changes, *GBA1* status, and fluctuations in cognitive and motor function, suggesting its relevance to PD progression. These findings underscore the potential of neuropsychiatric profiles as early indicators of PD risk, warranting further investigation.

## Methods

### Cohort

The UK Biobank is a large-scale national cohort study that recruited over 500,000 participants across the UK between 2006 and 2010, with its methodological details described elsewhere^67^. Data were extracted on 24th February 2024 (UKB project ID: 78867). HC were identified by excluding individuals with common neurological and psychiatric conditions (see Supplementary Materials, Table S1 for a full list), resulting in 324,619 HC participants, of whom 93,989 had sufficient data for factor analysis (Table S3, Fig. S1). Additionally, we initially identified 4571 participants with PD and 36,875 with CVD, including prevalent cases at the time of mental health questionnaire assessment as well as future incident cases. The final sample sizes used for analysis—696 for PD (of which 289 were prevalent cases) and 6560 for CVD (of which 3759 were prevalent)—were determined based on the feasibility of conducting factor analysis (see Methods, Factor Analysis section), and are summarised in Table 1. All participants provided written informed consent, and the study was approved by the Research Ethics Committee (REC number 16/NW/0274).

### Neuropsychiatric data

Neuropsychiatric data were extracted from two key assessments:

- **Mental health questionnaire (MHQ1)**: Introduced in July 2016, MHQ1 was designed to capture a broad spectrum of self-reported mental health symptoms, lifetime psychiatric diagnoses, psychological traits, and trauma-related experiences.
- **Mental well-being follow-up questionnaire (MHQ2)**: Launched in October 2022, MHQ2 aimed to address emerging mental health challenges following the COVID-19 pandemic. This follow-up questionnaire expanded upon MHQ1 by incorporating additional measures of resilience, well-being, and psychiatric symptoms such as panic disorder and eating disorders. Participants who completed either or both questionnaires were included in the study. The neuropsychiatric domains included in the study and factor analysis:
- **Depression severity:** Patient Health Questionnaire (PHQ-9), assessing depressive symptoms over the past two weeks.
- **Anxiety symptoms:** Generalised Anxiety Disorder Assessment (GAD-7), evaluating anxiety symptoms over the past two weeks.
- **Lifetime psychiatric disorders:** Composite international diagnostic interview short form (CIDI-SF), including subscales for depression severity, anxiety disorders, mania, and psychotic symptoms.
- **Post-traumatic stress symptoms:** PTSD Checklist (PCL-S), measuring symptoms related to trauma exposure.
- **Alcohol-use patterns:** Alcohol-Use Disorders Identification Test (AUDIT), assessing alcohol consumption and associated behaviours.
- **Trauma measures:** Childhood Trauma Screener (CTS-5), capturing adverse childhood experiences such as neglect or abuse. Additional measures assessed adverse adult experiences related to financial insecurity and social relationships.
- **Resilience:** Items evaluating psychological recovery and adaptability in response to stress or adversity.
- **Self-harm:** Self-reported behaviours related to intentional self-injury.
- **Subjective well-being:** Measures of life satisfaction and happiness.
- **Loneliness:** Frequency of social isolation or feelings of loneliness.
- **Addiction behaviours:** Lifetime and ongoing addiction-related behaviours, including substance use and behavioural addictions.

The total scores and subscores of these measures were derived from standardised scales embedded within the MHQ1 and MHQ2 questionnaires.

The extracted data underwent systematic processing to prepare for analyses. Reverse coding was applied to a few items within the trauma and resilience scales to ensure consistency in scoring across measures. For example, items such as *”Loved as a child”* in the Childhood Trauma Screener (CTS-5) were reverse-coded so that higher scores reflected greater trauma exposure. Similarly, items such as *”Bounce back after hard times”* in the resilience scale were reverse-coded so that higher scores indicated greater resilience.

Composite scores for each measure were calculated by summing individual item responses within each scale. For example, total scores for PHQ-9, GAD-7, CIDI-SF subscales (e.g., depression severity, anxiety disorders), PCL-S, AUDIT, resilience, trauma exposure, self-harm behaviours, subjective well-being, loneliness, addiction-related measures, and psychotic experiences were computed to provide summary indices of each construct. However, factor analysis was conducted at the item level, rather than the total scores.

### Neuroimaing data

MRI data acquisition in the UK Biobank was done using a Siemens Skyra 3.0.T scanner (Siemens Medical Solutions, Germany) with a 32-channel head coil. T1-weighted images (1 mm^3^ isotropic resolution) were analysed using the FMRIB Software Library (FSL) (http://fsl.fmribox.ac.uk/fsl.), and image-derived phenotypes (IDPs), including total brain and regional grey matter volumes, were made publicly available. Detailed MRI acquisition and analysis protocols have been described elsewhere^68,69^. We used previously validated IDPs of volumetric and susceptibility-weighted MRI (swMRI) (average values for each group reported in Table S6). Volume estimates were corrected for head size using a head-scaling variable derived from the MRI scan, which accounts for volumetric transformation to standard space based on the outer-skull surface.

swMRI analysis included T2* scores and QSM, providing complementary information about tissue composition and microstructure. T2* reflects signal decay due to spin-spin interactions and local magnetic field inhomogeneities, making it sensitive to microscopic variations in iron, myelin, and calcifications. Shorter T2* values indicate increased magnetic susceptibility variations, often associated with neurodegenerative processes^69^. In contrast, QSM quantifies tissue magnetic susceptibility (*χ*), providing a direct measure of bulk iron and myelin content. While paramagnetic substances (e.g., iron) and diamagnetic substances (e.g., myelin) have opposite effects on *χ* in QSM, they exert similar effects on T2*^69^. QSM has been used to detect disease-relevant changes, such as iron accumulation in neurodegenerative disorders, and to characterise microstructural alterations in ageing^70–72^.

As PD is characterised by significant subcortical brain changes, particularly in the early stages^73–76^, this study focuses on key subcortical regions, including the nucleus accumbens, amygdala, caudate, hippocampus, pallidum, putamen, thalamus, and substantia nigra. Measures were averaged across bilateral regions, yielding 24 imaging-derived phenotypes (IDPs) encompassing volume, QSM, and T2* values.

### Genetic data

*GBA1* carrier status was determined based on the presence of any *GBA1* risk alleles (*rs*76763715_*C*_, *rs*75548401_*A*_, *rs*2230288_*T*_), regardless of the number of copies. To ensure the specificity of the results to *GBA1*, APOE ε4 carrier status was also examined in the same models that included *GBA1* carrier status.

### Cognitive and motor data

Executive function is one of the primary cognitive domains affected in PD^59^. A single measure of executive function was computed using CFA based on four executive function tasks: snap Reaction Time, trail-Making Task, digit symbol substitution test, and tower rearranging task. This approach aligns with previous studies^77,78^ (See Supplementary Materials); however, pair matching was not included in the executive function score, as it is often regarded as a memory task^79^.

Two measures of motor function were analysed. The first was average handgrip strength, and the second was a composite score of motor symptoms derived from hospital episode statistics (HES) data. The HES-based measure included recorded instances of bradykinesia, tremor, rigidity, falls, balance impairment, and gait disturbances. However, we acknowledge that this measure may lack specificity to PD and may not exhibit a clear temporal correlation with the study variables.

For meaningful temporal correlations, only data from the biobank third assessment session were included, as this session was closest in time to when the MHQ and MRI data were obtained (time difference in years between MHQ and session 3: *µ* = *−*1.69*, SD* = 2.66). However, a longitudinal within-participant analysis (from baseline assessment (visit 1) to third session assessment (visit 3)) was conducted for reaction time and grip strength data. Reaction time was the only measure recorded at baseline.

### Factor analysis

Factor analysis was conducted to identify latent variables within the control group dataset across eligible items (51 in total) from 11 different questionnaires in the MHQ1 (raw data had 17 questionnaires, Fig. 1A). These were included after exclusion of items from MHQ1 and MHQ2 with excessive missing values. Only complete cases were retained, and variables with zero variance were removed to ensure meaningful analysis (HC *N* = 93,898). The threshold for missingness (70,000 observations for HC) and the number of variables retained (51) were determined based on a trade-off analysis that balanced sample size with variable coverage for the three different groups. Specifically, information scores were calculated by multiplying the number of retained variables for each missingness threshold by the sample size (normalised across thresholds), allowing for the identification of the optimal threshold that maximised informational content while maintaining sufficient data completeness (see Fig. S1 and Table S3).

To assess factorability, the Kaiser-Meyer-Olkin (KMO) test was performed to measure sampling adequacy (*KMO* > 0.8 for all groups). Bartlett’s test of sphericity was used to determine whether the correlation matrix was suitable for factor analysis. A scree plot was generated to visually assess the optimal number of factors (Fig. 1B).

Following these preparatory steps, factor analysis was implemented in R (vf4.1.1) with *fa* function using an oblique (oblimin) rotation with four predefined factors. The same steps were repeated for PD and CVD. To evaluate factor congruence between the groups, the factor loadings from the control group were compared to those of the CVD and PD groups using Procrustes rotation (Fig. 1C).

To compute consistent factor scores across the groups, factor score coefficients were obtained by computing the inverse of the correlation matrix and applying it to the factor loadings from the control group. Standardised PD and CVD item data were then multiplied by these coefficients to derive individual factor scores. This methodological approach ensured consistency in factor structure assessment across the control, PD, and CVD groups, facilitating robust comparisons of latent factor distributions.

### Statistical analyses

All statistical tests were conducted in R (v4.1.1) or MATLAB v2024b. Multiple regression models were performed using *figlme* function in MATLAB or *lm* function in R, controlling for age, gender, and education in all models. Additional covariates were also included depending on the test (e.g., MRI imaging centre and time difference between MHQ assessment and imaging acquisition) for analysis involving MRI data. Statistical details along with model descriptions are reported in the Supplementary Materials. This research utilised Queen Mary’s *Apocrita* HPC facility, supported by QMUL Research-IT. 10.5281/zenodo.438045.

The relationship between key variables and age or time since diagnosis was assessed using a sliding window approach with fixed age-quantile widths moved along the age distribution (using a 20% Gaussian kernel code: conditionalPlot.m available: https://osf.io/vmabg/). This approach has been validated in multiple previous reports^78,80^, and offers methodological advances in modelling non-linearity. All variables plotted with the approach were controlled for age at measure.

Time (years) since diagnosis is calculated as the difference between the age of completion of MHQ1 and the earliest report of diagnosis age, whether obtained from self-report, HES or GP records.

Multiple comparisons were corrected for using the Bonferroni method and reported accordingly with the results.

## Supplementary information

Supplementary Information

## Data Availability

The data used in this study are not publicly available and were accessed via the UK Biobank (\url{https://www.ukbiobank.ac.uk/}). They include demographic, clinical, and neuroimaging information from over 500,000 UK participants. Access is available to registered researchers with approved health-related projects that serve the public interest.
