# Supplementary material for: The relationship between neuropsychiatric dimensions and markers of Parkinson’s disease risk in the UK Biobank
Source: NPJ Parkinsons Dis. 2025 Dec 1;11:344. doi: 10.1038/s41531-025-01181-y (PMC12669592; doi:10.1038/s41531-025-01181-y)
Supplement: Supplementary file 1 — Supplementary Information [file 41531_2025_1181_MOESM1_ESM.pdf]

Supplementary Materials

Confirmatory Factor Analysis of Executive Function

A confirmatory factor analysis (CFA) was conducted to test a single latent factor model of *Executive Function*, derived from four cognitive performance tasks: Snap Reaction Time, Trail Making, Digit Symbol Substitution, and Tower Rearranging. The model was estimated using maximum likelihood (ML) estimation in the `lavaan` package (version 0.6.15; Rosseel, 2012) in R. All four indicators were specified to load on a single latent factor, without cross-loadings or correlated residuals.

Cognitive Measures. The indicators reflected a range of executive abilities. Snap Reaction Time measured response speed, recorded in seconds, where higher values indicate slower responses and hence poorer performance. Trail Making was defined as the difference in response times between the more complex condition B and the simpler condition A, with higher scores indicating worse performance. The Digit Symbol Substitution task measured response accuracy, where higher scores denote better performance. Similarly, the Tower Rearranging task assessed planning accuracy, with higher values also indicating better performance.

Sample and Estimation. The analysis included $N = 57{,}271$ participants who completed the tests in the larger UK Biobank sample. A total of 12 parameters were estimated using the NLMINB optimisation algorithm. There were five patterns of missingness in the dataset, which were handled using full information maximum likelihood estimation.

Model Fit. The model showed excellent fit to the data:

- $\chi^2 = 56.38, p < .001$
- Comparative Fit Index (CFI) = 0.998
- Tucker–Lewis Index (TLI) = 0.993
- Root Mean Square Error of Approximation (RMSEA) = 0.022, 90% CI [0.017, 0.027], $p_{\text{close}} = 1.000$

- Standardised Root Mean Square Residual (SRMR) = 0.007

Standardised Factor Loadings. All observed variables loaded significantly on the latent *Executive Function* factor:

- Snap Reaction Time: $\lambda = 0.398$, $SE = 0.005$, $z = 83.97$, $p < .001$
- Trail Making: $\lambda = 0.565$, $SE = 0.005$, $z = 120.13$, $p < .001$
- Digit Symbol Substitution: $\lambda = 0.687$, $SE = 0.005$, $z = 142.67$, $p < .001$
- Tower Rearranging: $\lambda = 0.538$, $SE = 0.005$, $z = 103.17$, $p < .001$

Residual Variances. Standardised residual variances were moderate to high:

- Snap Reaction Time: 0.842
- Trail Making: 0.681
- Digit Symbol Substitution: 0.528
- Tower Rearranging: 0.711

Intercepts. The standardised intercepts of the observed indicators were:

- Snap Reaction Time: -0.023, $p < .001$
- Trail Making: -0.005, $p = .218$
- Digit Symbol Substitution: 0.009, $p = .026$
- Tower Rearranging: -0.012, $p = .017$

Latent Factor Properties. The latent *Executive Function* factor was standardised, with its variance fixed to 1.0 and mean fixed to 0 for model identification.

These results provide strong empirical support for the unidimensional structure of executive function, with all indicators contributing significantly to the latent construct and the model demonstrating robust global fit.

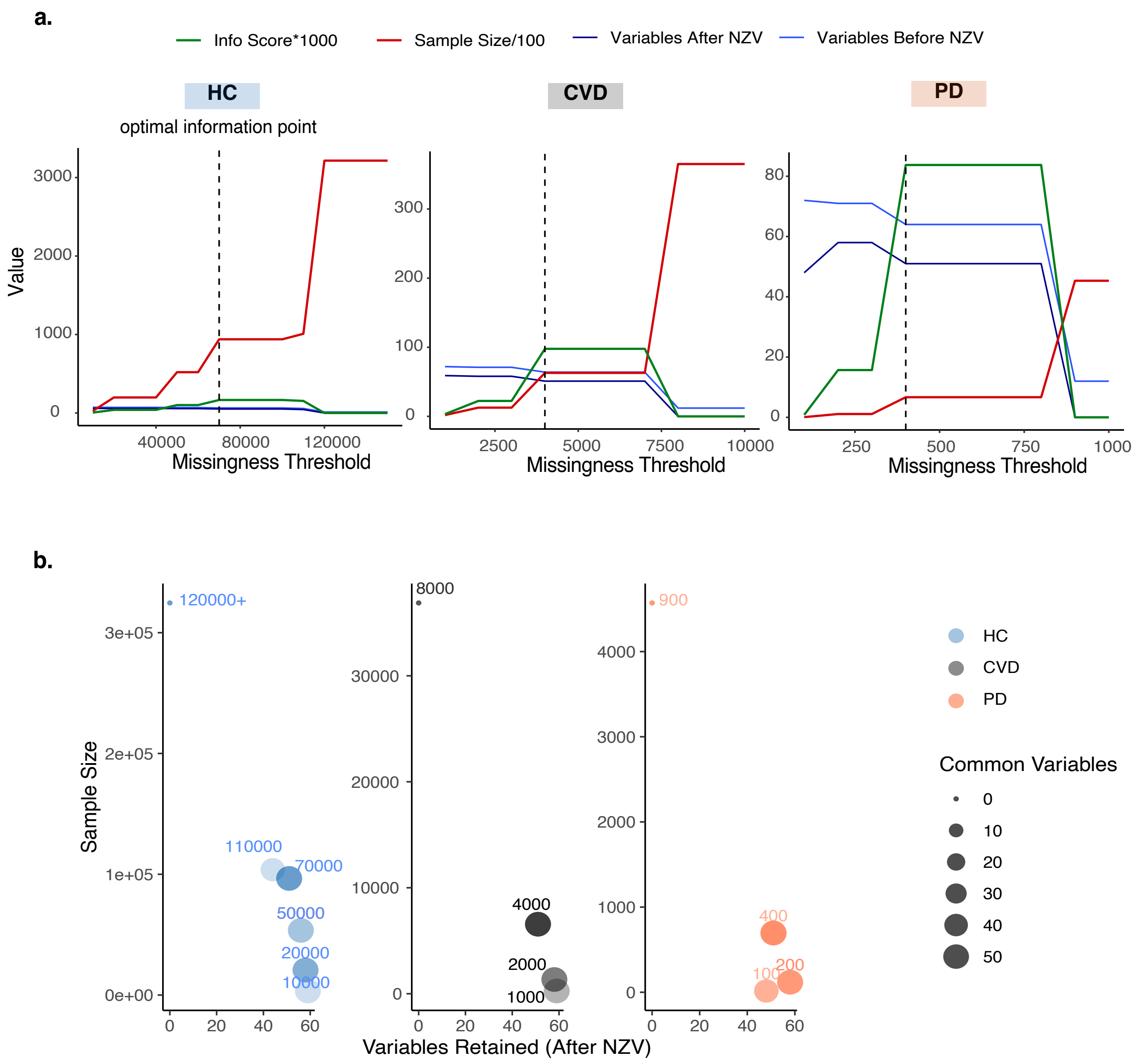

Figure S1: **Selection of datasets and thresholds based on the trade-off between sample size and variable retention.** **a.** Relationship between missingness thresholds and the number of variables retained after removing zero-variance variables and restricting to complete cases. To identify the optimal threshold for each dataset, an information score was computed as the number of retained variables multiplied by the sample size (normalised across thresholds). This score facilitated selection of the threshold that best balances data completeness and dimensionality (see Table S3). **b.** Trade-off analysis illustrating the relationship between retained variables, sample size, and shared variables across datasets. At the selected thresholds, 51 variables were found to be common to all three datasets, providing a robust basis for subsequent factor analysis. NZV: non-zero variance.

Condition Category	Reference Code
Anxiety Disorders	ICD-10 (f41270 codes F41, F410, F411, F412, F413, F418, F419), F064
Apathy	ICD-10 (f41270 code R453)
Behavioural and Physiological Disturbances	ICD-10 (f41270 code F59)
Bipolar Disorder	ICD-10 (F41270 codes F31, F310–F319, F30, F300–F302, F308, F309),<br>SR (F200002 code 1291)
Brain Damage	ICD-10 (f41270 codes F06, F060–F069)
Brain Infections	ICD-10 (F41270 codes G92, G00–G08, A81, A83–A89, A17, A20, A32, A50, A52,<br>B00–B02, B05–B06, B22, B26, B37, B38, B91, B94, I67)
Brain Tumours	ICD-10 (F41270 codes C71–C72, D33, D43)
Cerebral Palsy	ICD-10 (F41270 code G80), SR (F200002 code 1433)
Delirium	ICD-10 (F41270 code F05)
Delusional Disorders	ICD-10 (F41270 codes F22, F24)
Dementia	FO (F130840, F130842, F130836, f131036, f130838),<br>ADO (F42018, F42024, F42020, F4202),<br>ICD-10 (F41270 codes F000, F001, F002, F009, G300, G301, G308, G309, F01, F010,<br>F011, F012, F013, F018, F019, A810, F020, F021, F022, F023, F024, F028, F03,<br>F051, G310, G311, G318, I673), SR (F200002 code 1263)
Demyelinating Diseases	ICD-10 (f41270 codes G35–G37), SR (F200002 codes 1261, 1397)
Depression	FO (f130896), ADO (f130894),<br>ICD-10 (f41270 codes f32, F33, f34, f38, f339), SR (f200002 code 1268)
Guillain-Barré Syndrome	FO(f131084), ICD(G61), SR(F200002 code 1256)
Head Injury	ICD-10(f130894 codes S02-09, S140, S240, S430, f072, T90), SR (F200002 codes 1240, 1266, 1626)
Huntington's Disease	ICD-10 (f130894 codes F41270 code G10)
Hydrocephalus	ICD-10 (f41270 codes G91, G94)
Mental and Behavioural Disorders (Substance Use)	FO(F130854, F130856, F130858, F130860, F130862, F130864, F130866, F130868, F130870, F130872),<br>ICD (f130894 codes F42170, F10-F19)
Obsessive-Compulsive Disorder (OCD)	FO (F130908), ICD-10(f41270 codes F42)
Neurodegenerative Disorders	ICD-10 (f41270 code G122), SR (F200002 code 1259),<br>ICD-10 (f41270 codes G35–G37), SR (F200002 codes 1261, 1397),
Myasthenia Gravis	FO (131092), ICD-10 (G70), SR (F200002 codes 1260, 1437)
Parkinson's Disease	FO (F131022), ADO (F42032),<br>ICD-10 (f41270 code G20), SR (F200002 code 1262),
Parkinsonism	FO (F13014, F13130, F131032, F131024, f13026, F13028, F42030, F42034, F42036)<br>ICD-10 (f41270 code G21-G25, G11), SR (F200002 codes 1525)
Personality Disorders	ICD-10 (f41270 code F07)
Psychosis and Schizophrenia Spectrum	FO (F130884, 130874, 130876), ICD-10 (f41270 codes F20, F21, F23, F28, F25),<br>SR (F200002 code 1289),
Cerebrovascular Disease	FO (F42006, F42008, F42010, F42012, F131056, F131058, F131360, F131362,<br>F131364, F131366, F131368,x F131370, F131372, F131374, F131376, F131378)<br>ICD-10 (f41270 codes G45, G46, I60-I69)<br>SR (F200002 codes 108, 1082, 1083, 1086, 1491, 1491, 1425, 1583)

Table S1: **Exclusion criteria for defining healthy controls based on specific conditions and their reference codes.** ADO (algorithmically-defined outcomes), FO (first occurrence), SR (self-report).

Measure	IDs
PHQ-9 (Depression Severity)	F20514, F20510, F20517, F20519, F20511, F20507, F20508, F20518, F20513
CIDI Depression Severity	F20446, F20441, F20532, F20435, F20449, F20450, F20436, F20439, F20440, F20442, F20534, F20437, F20546, F20533, F20535, F20536
CIDI Mania Severity	F20502, F20501, F20492, F20548, F20493
GAD-7 (Anxiety Severity)	F20506, F20509, F20520, F20515, F20516, F20505, F20512
CIDI-SF Anxiety	F20421, F20420, F20425, F20542, F20538, F20543, F20541, F20540, F20539, F20537, F20426, F204723, F20429, F20419, F20422, F20417, F20427, F20428, F20550, F20418
Addiction (Lifetime)	F20401, F20406, F20404, F20503, F20504, F20456, F20431
Addiction (Ongoing)	F20415, F20457, F20432
AUDIT (Alcohol Use)	F20414, F20403, F20416, F20413, F20407, F20412, F20409, F20408, F20411, F20405
Cannabis Use	F20454
CIDI Psychosis	F20471, F20473, F20463, F20465, F20474, F20476, F20468, F20470, F20467, F20461, F20462, F20477, F20466
Childhood Trauma (CTS-5)	F20489, F20488, F20487, F20490, F20491
Adverse Adult Experiences	F20522, F20523, F20521, F20524, F20525, F20531, F20529, F20526, F20530, F20528, F20527
PTSD (PCL-S)	F20497, F20498, F20495, F20496, F20494
Self-Harm	F20479, F20485, F20486, F20480, F20482, F20481, F20553, F20483, F20484
Subjective Well-being	F20458, F20459, F20460
Loneliness	F29171, F29172, F29173, F29174
Resilience	F29175, F29176, F29177, F29178, F29179, F29180
Cognitive Function (Executive Tasks)	F20023 (Reaction Time), F6350, F6348 (Trail Making), F23324 (Digit Symbol), F21004 (Tower Rearranging)
Motor Function (Hand Grip)	F46 (Left), F47 (Right)
Bradykinesia (ICD-10)	F41270 (R258)
Falls (ICD-10)	F41270 (W01, W18, W10, W17, W06–W08, W19)
Balance Difficulties (ICD-10)	F41270 (R26, R27)
Tremor (ICD-10)	F41270 (G25)
Rigidity (ICD-10)	F41270 (R298, W628)

Table S2: **Neuropsychiatric, cognitive, motor, and Parkinson's-related symptom measures with corresponding UK Biobank field IDs and ICD-10 codes.**

Dataset	Threshold	Variables above threshold	Variables with no zero-variance	Sample Size
HC	10000	72	59	2,533
HC	20000	71	58	19,781
HC	30000	71	58	19,781
HC	40000	71	58	19,781
HC	50000	69	56	51,977
HC	60000	69	56	51,977
HC	70000	64	51	93,898
HC	80000	64	51	93,898
HC	90000	64	51	93,898
HC	100000	64	51	93,898
HC	110000	57	44	100,902
HC	120000	12	0	321,547
PD	100	72	48	7
PD	200	71	58	110
PD	300	71	58	110
PD	400	64	51	668
PD	500	64	51	668
PD	600	64	51	668
PD	700	64	51	668
PD	800	64	51	668
PD	900	12	0	4,533
CVD	1000	72	59	185
CVD	2000	71	58	1,263
CVD	3000	71	58	1,263
CVD	4000	64	51	6,290
CVD	5000	64	51	6,290
CVD	6000	64	51	6,290
CVD	7000	64	51	6,290
CVD	8000	12	0	36,512

Table S3: **Missing data threshold analysis results.** This table presents the effects of applying different missingness thresholds across three datasets (HC, PD, and CVD). For each threshold value, the table reports the number of variables retained that have observations above the threshold, the number of variables after removing zero-variance columns, and the final sample size after applying complete case analysis. Higher thresholds result in fewer variables but larger sample sizes, while lower thresholds retain more variables at the cost of reduced sample size due to listwise deletion. These results facilitate the identification of optimal thresholds that balance variable retention with adequate sample size for subsequent factor analysis. A threshold of 70000, 400 and 4000 was chosen for HC, PD and CVD, respectively, as this achieves a reasonable sample size across groups with consistent variables. Note that the sample size reported in Table 1 is higher for PD and CVD as the complete case analysis was applied on 51 variables identified with HC, rather than the 64 variables that would have been otherwise used after threshold application in this table.

Variable	Years Bin	PD	CVD
Factor Scores	$-5 \leq x < 0$	343	2466
	$0 \leq x < 5$	143	1354
	$5 \leq x < 10$	57	927
	$10 \leq x < 15$	62	673
Executive Function	$-5 \leq x < 0$	38	387
	$0 \leq x < 5$	11	270
	$5 \leq x < 10$	10	163
	$10 \leq x < 15$	3	101
Hand Grip	$-5 \leq x < 0$	49	563
	$0 \leq x < 5$	21	372
	$5 \leq x < 10$	16	238
	$10 \leq x < 15$	6	151
PD Symptoms	$-5 \leq x < 0$	343	2466
	$0 \leq x < 5$	143	1354
	$5 \leq x < 10$	57	927
	$10 \leq x < 15$	62	673

Table S4: Number of participants in PD and CVD groups with available data for each variable across bins of years since diagnosis (x).

	Factor 1	Factor 2	Factor 3	Factor 4
(Intercept)	$\beta = +0.727$<br>$SE = 0.0444$<br>$t_{99530} = +16.38$<br>***p<0.0001***	$\beta = +0.0661$<br>$SE = 0.0445$<br>$t_{99530} = +1.49$<br>$p = 0.14$	$\beta = +0.0752$<br>$SE = 0.0349$<br>$t_{99530} = +2.15$<br>***p = 0.031***	$\beta = -0.518$<br>$SE = 0.0331$<br>$t_{99530} = -15.64$<br>***p<0.0001***
Age	$\beta = -0.094$<br>$SE = 0.00375$<br>$t_{99530} = -25.06$<br>***p<0.0001***	$\beta = -0.0326$<br>$SE = 0.00376$<br>$t_{99530} = -8.67$<br>***p<0.0001***	$\beta = -0.0535$<br>$SE = 0.00295$<br>$t_{99530} = -18.12$<br>***p<0.0001***	$\beta = -0.172$<br>$SE = 0.0028$<br>$t_{99530} = -61.63$<br>***p<0.0001***
Education	$\beta = -0.0122$<br>$SE = 0.0037$<br>$t_{99530} = -3.31$<br>***p = 0.00095***	$\beta = -0.0084$<br>$SE = 0.00371$<br>$t_{99530} = -2.27$<br>***p = 0.023***	$\beta = +0.0064$<br>$SE = 0.00291$<br>$t_{99530} = +2.20$<br>***p = 0.028***	$\beta = +0.000692$<br>$SE = 0.00276$<br>$t_{99530} = +0.25$<br>$p = 0.80$
Gender	$\beta = +0.164$<br>$SE = 0.00742$<br>$t_{99530} = +22.12$<br>***p<0.0001***	$\beta = -0.25$<br>$SE = 0.00744$<br>$t_{99530} = -33.55$<br>***p<0.0001***	$\beta = -0.233$<br>$SE = 0.00584$<br>$t_{99530} = -39.92$<br>***p<0.0001***	$\beta = +0.538$<br>$SE = 0.00554$<br>$t_{99530} = +97.09$<br>***p<0.0001***
CVD vs PD	$\beta = -0.453$<br>$SE = 0.0464$<br>$t_{99530} = -9.77$<br>***p<0.0001***	$\beta = -0.0341$<br>$SE = 0.0464$<br>$t_{99530} = -0.73$<br>$p = 0.46$	$\beta = +0.222$<br>$SE = 0.0365$<br>$t_{99530} = +6.09$<br>***p<0.0001***	$\beta = +0.218$<br>$SE = 0.0346$<br>$t_{99530} = +6.30$<br>***p<0.0001***
HC vs PD	$\beta = -0.804$<br>$SE = 0.0443$<br>$t_{99530} = -18.14$<br>***p<0.0001***	$\beta = +0.0438$<br>$SE = 0.0444$<br>$t_{99530} = +0.99$<br>$p = 0.32$	$\beta = +0.0262$<br>$SE = 0.0349$<br>$t_{99530} = +0.75$<br>$p = 0.45$	$\beta = +0.271$<br>$SE = 0.0331$<br>$t_{99530} = +8.19$<br>***p<0.0001***
$adj - R^2$	0.02	0.01	0.02	0.11
N_{obs}	99536	99536	99536	99536
BIC	312256.03	312656.19	264520.90	253948.71

Table S5: **Effect of group on factor scores.** Models were specified as follows. Factor Score $\sim$ 1 + group + gender + Education + Age. HC: Healthy Controls. PD: Parkinson's Disease. CVD: Cerebrovascular Disease.

Region / Measure	CVD	HC	PD
**QSM**			
Thalamus	-10.642	-11.670	-9.4303
Caudate	32.005	29.335	32.235
Putamen	32.657	27.682	32.913
Pallidum	78.728	77.283	78.326
Hippocampus	-4.7455	-6.4428	-5.4543
Amygdala	-7.143	-8.0228	-6.6904
Accumbens	-7.5114	-7.3356	-8.149
SubstantiaNigra	66.681	64.550	73.947
**T2***			
Accumbens	40.677	40.903	41.470
Amygdala	44.995	45.537	45.367
Caudate	41.581	42.535	41.104
Hippocampus	45.003	45.539	45.277
Pallidum	28.214	28.574	28.333
Putamen	35.954	37.100	35.738
Thalamus	46.482	46.421	46.648
SubstantiaNigra	32.349	32.698	31.210
**Volume (mm^3)**			
Accumbens	516.61	564.83	495.94
Amygdala	1578.4	1604.2	1583
Caudate	4408.9	4467.4	4304.2
Hippocampus	4710.2	4942	4587.4
Pallidum	2204.4	2290.9	2242.1
Putamen	5966.4	6165.4	5844.4
Thalamus	9501.1	9862.2	9478.3

Table S6: **QSM, T2*, and volumetric measures for HC, PD, and CVD groups.**

Variable	Factor 1			Factor 2			Factor 3			Factor 4		
	Correlation	p-value	Adj. p-value	Correlation	p-value	Adj. p-value	Correlation	p-value	Adj. p-value	Correlation	p-value	Adj. p-value
**QSM**												
Thalamus	0.001582	0.174675	1.000000	-0.000533	0.649045	1.000000	0.000064	0.945050	1.000000	-0.005168	0.000000	0.000002
Caudate	0.000102	0.875403	1.000000	0.000099	0.879249	1.000000	0.000765	0.137630	1.000000	0.004130	0.000000	0.000000
Putamen	0.000121	0.820920	1.000000	0.000198	0.711884	1.000000	0.000806	0.056077	1.000000	0.004906	0.000000	0.000000
Pallidum	0.001011	0.041010	1.000000	-0.000788	0.112810	1.000000	0.001712	0.000012	0.001139	-0.001004	0.010147	0.933536
Hippocampus	0.001646	0.235226	1.000000	-0.000664	0.633667	1.000000	0.000926	0.399032	1.000000	0.002699	0.013599	1.000000
Amygdala	-0.000428	0.706497	1.000000	0.000505	0.657724	1.000000	0.000685	0.446392	1.000000	-0.000342	0.702467	1.000000
Accumbens	-0.000723	0.430406	1.000000	0.000740	0.421675	1.000000	-0.000772	0.287769	1.000000	0.001040	0.150587	1.000000
SubstantiaNigra	0.001049	0.043163	1.000000	-0.000907	0.081664	1.000000	0.001127	0.006052	0.556826	0.001647	0.000057	0.005266
**T2***												
Accumbens	-0.003279	0.026463	1.000000	0.001272	0.389493	1.000000	0.000133	0.908795	1.000000	0.001801	0.120791	1.000000
Amygdala	-0.008329	0.000007	0.000601	0.003923	0.033765	1.000000	-0.002692	0.064614	1.000000	0.002156	0.137580	1.000000
Caudate	-0.005320	0.012503	1.000000	0.003446	0.105728	1.000000	-0.002468	0.141652	1.000000	-0.011016	0.000000	0.000000
Hippocampus	-0.010979	0.000002	0.000157	0.005479	0.016964	1.000000	-0.005523	0.002266	0.208462	-0.002182	0.226151	1.000000
Pallidum	-0.003817	0.134372	1.000000	0.002236	0.380468	1.000000	-0.003795	0.058989	1.000000	0.000415	0.835885	1.000000
Putamen	-0.002702	0.155319	1.000000	0.001655	0.384120	1.000000	-0.001335	0.373173	1.000000	-0.013702	0.000000	0.000000
Thalamus	-0.001703	0.560287	1.000000	0.000848	0.771890	1.000000	0.002236	0.332104	1.000000	-0.001419	0.536818	1.000000
SubstantiaNigra	-0.004426	0.043896	1.000000	0.004010	0.069133	1.000000	-0.001811	0.297671	1.000000	-0.003038	0.079616	1.000000
**Volume**												
accumbens	-0.000110	0.103576	1.000000	0.000061	0.365243	1.000000	-0.000193	0.000289	0.026617	-0.000251	0.000002	0.000209
amygdala	-0.000063	0.034973	1.000000	0.000022	0.447902	1.000000	0.000021	0.381520	1.000000	0.000000	0.994393	1.000000
caudate	-0.000007	0.705634	1.000000	0.000026	0.127945	1.000000	0.000011	0.432086	1.000000	-0.000031	0.023171	1.000000
hippocampus	-0.000040	0.007060	0.649531	0.000018	0.210006	1.000000	0.000004	0.729293	1.000000	-0.000089	0.000000	0.000000
pallidum	-0.000050	0.082354	1.000000	0.000049	0.084353	1.000000	-0.000034	0.139534	1.000000	-0.000126	0.000000	0.000002
putamen	-0.000019	0.150074	1.000000	0.000014	0.266874	1.000000	-0.000021	0.039193	1.000000	-0.000054	0.000000	0.000020
thalamus	-0.000024	0.031118	1.000000	0.000018	0.096163	1.000000	-0.000028	0.001451	0.133464	-0.000086	0.000000	0.000000

Table S7: **Subcortical MRI correlations with factor scores across Groups**. QSM: Quantitative susceptibility mapping. Volumes are adjusted for intracranial volume. Correlations are controlled for age, gender, education, age difference between age at MRI acquisition and age at questionnaire completion, and assessment centre. p-value adjusted using the Bonferroni method (92 comparisons).

	SN QSM	Hippocampus volume
(Intercept)	$\beta = +72.2$	$\beta = +4.85 \times 10^{+03}$
	$SE = 2.04$	$SE = 61.1$
	$t_{17910} = +35.41$	$t_{19951} = +79.26$
	p<0.0001	***p<0.0001***
Age	$\beta = +0.456$	$\beta = -182$
	$SE = 0.123$	$SE = 3.83$
	$t_{17910} = +3.71$	$t_{19951} = -47.48$
	p = 0.00021	***p<0.0001***
Education	$\beta = +0.0603$	$\beta = -7.65$
	$SE = 0.126$	$SE = 4$
	$t_{17910} = +0.48$	$t_{19951} = -1.91$
	$p = 0.63$	$p = 0.06$
Gender	$\beta = +2.65$	$\beta = -291$
	$SE = 0.237$	$SE = 7.53$
	$t_{17910} = +11.16$	$t_{19951} = -38.58$
	p<0.0001	***p<0.0001***
CVD vs PD	$\beta = -6.9$	$\beta = +102$
	$SE = 2.05$	$SE = 63$
	$t_{17910} = -3.36$	$t_{19951} = +1.62$
	p = 0.00077	$p = 0.11$
HC vs PD	$\beta = -8.55$	$\beta = +206$
	$SE = 1.99$	$SE = 61.1$
	$t_{17910} = -4.29$	$t_{19951} = +3.37$
	p<0.0001	***p = 0.00076***
$adj - R^2$	0.01	0.18
N_{obs}	17916	19957
BIC	149723.10	306914.70

Table S8: **Effect of froup (PD as base group) on subcortical measures.** Models were specified as follows. SN QSM $\sim$ 1 + Group + Gender + Education + Age + (1 |Centre); Hippocampus volume: Hippocampus volume $\sim$ 1 + Group + Gender + Education + Age + (1 |Centre).

	**Factor 1**	**Factor 2**	**Factor 3**	**Factor 4**
(Intercept)	$\beta = +0.294$<br>$SE = 0.184$<br>$t_{98360} = +1.60$<br>$p = 0.11$	$\beta = +0.106$<br>$SE = 0.00513$<br>$t_{98360} = +20.61$<br>***p<0.0001***	$\beta = +0.163$<br>$SE = 0.0568$<br>$t_{98360} = +2.88$<br>***p = 0.004***	$\beta = -0.349$<br>$SE = 0.065$<br>$t_{98360} = -5.38$<br>***p<0.0001***
APOE4 carrier	$\beta = -0.00388$<br>$SE = 0.0257$<br>$t_{98360} = -0.15$<br>$p = 0.88$	$\beta = +0.00413$<br>$SE = 0.0258$<br>$t_{98360} = +0.16$<br>$p = 0.87$	$\beta = -0.00821$<br>$SE = 0.0202$<br>$t_{98360} = -0.41$<br>$p = 0.68$	$\beta = -0.0331$<br>$SE = 0.0192$<br>$t_{98360} = -1.73$<br>$p = 0.08$
APOE4 carrier:Age	$\beta = -0.0321$<br>$SE = 0.0264$<br>$t_{98360} = -1.21$<br>$p = 0.22$	$\beta = +0.0212$<br>$SE = 0.0264$<br>$t_{98360} = +0.80$<br>$p = 0.42$	$\beta = -0.00234$<br>$SE = 0.0207$<br>$t_{98360} = -0.11$<br>$p = 0.91$	$\beta = -0.0101$<br>$SE = 0.0197$<br>$t_{98360} = -0.51$<br>$p = 0.61$
GBA1 carrier	$\beta = +0.0255$<br>$SE = 0.0179$<br>$t_{98360} = +1.42$<br>$p = 0.15$	$\beta = -0.00887$<br>$SE = 0.018$<br>$t_{98360} = -0.49$<br>$p = 0.62$	$\beta = -0.0404$<br>$SE = 0.0141$<br>$t_{98360} = -2.86$<br>***p = 0.0042***	$\beta = +0.015$<br>$SE = 0.0134$<br>$t_{98360} = +1.12$<br>$p = 0.26$
GBA1 carrier:Age	$\beta = +0.0147$<br>$SE = 0.0183$<br>$t_{98360} = +0.81$<br>$p = 0.42$	$\beta = -0.0182$<br>$SE = 0.0183$<br>$t_{98360} = -0.99$<br>$p = 0.32$	$\beta = +0.012$<br>$SE = 0.0144$<br>$t_{98360} = +0.83$<br>$p = 0.40$	$\beta = -0.0404$<br>$SE = 0.0136$<br>$t_{98360} = -2.96$<br>***p = 0.0031***
Age	$\beta = -0.0932$<br>$SE = 0.00389$<br>$t_{98360} = -23.96$<br>***p<0.0001***	$\beta = -0.0351$<br>$SE = 0.00386$<br>$t_{98360} = -9.10$<br>***p<0.0001***	$\beta = -0.0534$<br>$SE = 0.00306$<br>$t_{98360} = -17.45$<br>***p<0.0001***	$\beta = -0.17$<br>$SE = 0.0029$<br>$t_{98360} = -58.69$<br>***p<0.0001***
Education	$\beta = -0.0106$<br>$SE = 0.00372$<br>$t_{98360} = -2.86$<br>***p = 0.0043***	$\beta = -0.00826$<br>$SE = 0.00373$<br>$t_{98360} = -2.22$<br>***p = 0.027***	$\beta = +0.0062$<br>$SE = 0.00292$<br>$t_{98360} = +2.12$<br>***p = 0.034***	$\beta = +0.00161$<br>$SE = 0.00278$<br>$t_{98360} = +0.58$<br>$p = 0.56$
Gender	$\beta = +0.166$<br>$SE = 0.00747$<br>$t_{98360} = +22.17$<br>***p<0.0001***	$\beta = -0.252$<br>$SE = 0.00747$<br>$t_{98360} = -33.66$<br>***p<0.0001***	$\beta = -0.232$<br>$SE = 0.00587$<br>$t_{98360} = -39.59$<br>***p<0.0001***	$\beta = +0.538$<br>$SE = 0.00557$<br>$t_{98360} = +96.58$<br>***p<0.0001***
$adj - R^2$	0.02	0.01	0.02	0.11
N_{obs}	98368	98368	98368	98368
BIC	308688.63	309097.74	261268.85	251150.58

Table S9: **Effect of *GBA1* on factor scores.** Models were specified as follows. Factor Score $\sim$ 1 + gender + Education + *GBA1**Age + *APOE4**Age + (1 |group).

	**Executive Function**	**Hand Grip**	**Motor Symptoms**
(Intercept)	$\beta = -0.0353$	$\beta = +21.8$	$\beta = +0.183$
	$SE = 0.0463$	$SE = 0.414$	$SE = 0.0571$
	$t_{22415} = -0.76$	$t_{29070} = +52.74$	$t_{30034} = +3.20$
	$p = 0.45$	***p<0.0001***	***p = 0.0014***
Factor4	$\beta = +0.0347$	$\beta = +0.289$	$\beta = -7.33 \times 10^{-05}$
	$SE = 0.00505$	$SE = 0.0458$	$SE = 0.00178$
	$t_{22415} = +6.87$	$t_{29070} = +6.31$	$t_{30034} = -0.04$
	p<0.0001	***p<0.0001***	$p = 0.97$
Factor4:Age	$\beta = +0.0057$	$\beta = -0.208$	$\beta = -0.00304$
	$SE = 0.00496$	$SE = 0.0438$	$SE = 0.0017$
	$t_{22415} = +1.15$	$t_{29070} = -4.76$	$t_{30034} = -1.79$
	$p = 0.25$	***p<0.0001***	$p = 0.07$
Age	$\beta = -0.385$	$\beta = -2.4$	$\beta = +0.0194$
	$SE = 0.00475$	$SE = 0.043$	$SE = 0.00167$
	$t_{22415} = -81.01$	$t_{29070} = -55.94$	$t_{30034} = +11.60$
	p<0.0001	***p<0.0001***	***p<0.0001***
Age diff	$\beta = -0.0617$	$\beta = +0.173$	$\beta = +0.011$
	$SE = 0.00552$	$SE = 0.0414$	$SE = 0.00162$
	$t_{22415} = -11.18$	$t_{29070} = +4.18$	$t_{30034} = +6.81$
	p<0.0001	***p<0.0001***	***p<0.0001***
Education	$\beta = +0.11$	$\beta = +0.248$	$\beta = -0.00375$
	$SE = 0.0046$	$SE = 0.0408$	$SE = 0.00159$
	$t_{22415} = +23.97$	$t_{29070} = +6.07$	$t_{30034} = -2.36$
	p<0.0001	***p<0.0001***	***p = 0.018***
Gender	$\beta = +0.0621$	$\beta = +14.6$	$\beta = -0.0163$
	$SE = 0.00923$	$SE = 0.0836$	$SE = 0.00325$
	$t_{22415} = +6.72$	$t_{29070} = +174.56$	$t_{30034} = -5.02$
	p<0.0001	***p<0.0001***	***p<0.0001***
$adj - R^2$	0.27	0.56	0.05
N_{obs}	22422	29077	30041
BIC	44894.56	193866.28	6141.05

Table S10: **Effect of alcohol- and substance-related behaviours (Factor 4) on clinical attributes across groups.** Models were specified as follows. Dependent Variable $\sim$ 1 + Gender + Education + Age diff + Factor4*Age + (1 |Group). Age diff: Age difference in years between obtaining clinical measures and mental health questionnaire completion.

	Executive Function Before Dx	After Dx	Grip Strength (Before Dx) Grip Before Dx	After Dx
(Intercept)	$\beta = +0.0264$	$\beta = -0.268$	$\beta = +20.6$	$\beta = +21.7$
	$SE = 0.181$	$SE = 0.27$	$SE = 1.58$	$SE = 1.44$
	$t_{42} = +0.15$	$t_{23} = -0.99$	$t_{55} = +13.01$	$t_{42} = +15.12$
	$p = 0.88$	$p = 0.33$	***p<0.0001***	***p<0.0001***
Factor4	$\beta = -0.162$	$\beta = -0.474$	$\beta = +0.877$	$\beta = +0.471$
	$SE = 0.11$	$SE = 0.282$	$SE = 0.901$	$SE = 1.15$
	$t_{42} = -1.48$	$t_{23} = -1.68$	$t_{55} = +0.97$	$t_{42} = +0.41$
	$p = 0.15$	$p = 0.11$	$p = 0.33$	$p = 0.68$
Factor4:Age	$\beta = +0.326$	$\beta = -0.374$	$\beta = +1.15$	$\beta = -2.4$
	$SE = 0.136$	$SE = 0.211$	$SE = 1.08$	$SE = 1$
	$t_{42} = +2.40$	$t_{23} = -1.78$	$t_{55} = +1.06$	$t_{42} = -2.40$
	p = 0.021	$p = 0.09$	$p = 0.29$	***p = 0.021***
Age	$\beta = -0.351$	$\beta = -0.284$	$\beta = -0.933$	$\beta = -1.27$
	$SE = 0.112$	$SE = 0.166$	$SE = 0.88$	$SE = 0.901$
	$t_{42} = -3.15$	$t_{23} = -1.71$	$t_{55} = -1.06$	$t_{42} = -1.41$
	p = 0.003	$p = 0.10$	$p = 0.29$	$p = 0.17$
Age diff	$\beta = -0.0324$	$\beta = +0.707$	$\beta = +2.17$	$\beta = +0.995$
	$SE = 0.121$	$SE = 0.188$	$SE = 0.835$	$SE = 0.852$
	$t_{42} = -0.27$	$t_{23} = +3.76$	$t_{55} = +2.60$	$t_{42} = +1.17$
	$p = 0.79$	***p = 0.001***	***p = 0.012***	$p = 0.25$
Education	$\beta = -0.127$	$\beta = +0.328$	$\beta = +0.666$	$\beta = +0.168$
	$SE = 0.0955$	$SE = 0.218$	$SE = 0.774$	$SE = 0.857$
	$t_{42} = -1.33$	$t_{23} = +1.51$	$t_{55} = +0.86$	$t_{42} = +0.20$
	$p = 0.19$	$p = 0.15$	$p = 0.39$	$p = 0.85$
Gender	$\beta = -0.189$	$\beta = -0.197$	$\beta = +11.4$	$\beta = +11.2$
	$SE = 0.225$	$SE = 0.315$	$SE = 1.92$	$SE = 1.67$
	$t_{42} = -0.84$	$t_{23} = -0.62$	$t_{55} = +5.95$	$t_{42} = +6.72$
	$p = 0.41$	$p = 0.54$	***p<0.0001***	***p<0.0001***
$adj - R^2$	0.19	0.27	0.39	0.51
N_{obs}	49	30	62	49
BIC	132.52	93.74	434.67	332.25

Table S11: **Effect of alcohol- and substance-related behaviours (Factor 4) on clinical attributes in PD before and after Dx.** Models were specified as follows: Dependent Variable $\sim$ 1 + Gender + Education + Age diff + Factor4*Age.

	Grip Strength	Reaction Time
(Intercept)	$\beta = +25.5$<br>$SE = 0.0711$<br>$t_{59047} = +358.55$<br>***p<0.0001***	$\beta = +6.29$<br>$SE = 0.00167$<br>$t_{58317} = +3763.34$<br>***p<0.0001***
Age	$\beta = -2.15$<br>$SE = 0.0366$<br>$t_{59047} = -58.75$<br>***p<0.0001***	$\beta = +0.0522$<br>$SE = 0.000851$<br>$t_{58317} = +61.26$<br>***p<0.0001***
Education	$\beta = +0.186$<br>$SE = 0.0357$<br>$t_{59047} = +5.21$<br>***p<0.0001***	$\beta = -0.0079$<br>$SE = 0.000832$<br>$t_{58317} = -9.51$<br>***p<0.0001***
Gender	$\beta = +15.5$<br>$SE = 0.0721$<br>$t_{59047} = +215.19$<br>***p<0.0001***	$\beta = -0.0361$<br>$SE = 0.00167$<br>$t_{58317} = -21.56$<br>***p<0.0001***
Age diff	$\beta = -0.424$<br>$SE = 0.0348$<br>$t_{59047} = -12.19$<br>***p<0.0001***	$\beta = +0.00677$<br>$SE = 0.000808$<br>$t_{58317} = +8.39$<br>***p<0.0001***
Visit	$\beta = -3.45$<br>$SE = 0.0541$<br>$t_{59047} = -63.79$<br>***p<0.0001***	$\beta = +0.111$<br>$SE = 0.00138$<br>$t_{58317} = +80.12$<br>***p<0.0001***
Visit:CVD	$\beta = -0.51$<br>$SE = 0.173$<br>$t_{59047} = -2.95$<br>***p = 0.0032***	$\beta = +0.0121$<br>$SE = 0.00448$<br>$t_{58317} = +2.71$<br>***p = 0.0067***
Visit:HC (Below Factor 4 Median)	$\beta = +0.469$<br>$SE = 0.0794$<br>$t_{59047} = +5.91$<br>***p<0.0001***	$\beta = +0.00265$<br>$SE = 0.00203$<br>$t_{58317} = +1.30$<br>$p = 0.19$
Visit:PD	$\beta = -2.17$<br>$SE = 0.623$<br>$t_{59047} = -3.48$<br>***p = 0.0005***	$\beta = +0.0233$<br>$SE = 0.0166$<br>$t_{58317} = +1.40$<br>$p = 0.16$
CVD	$\beta = -0.467$<br>$SE = 0.179$<br>$t_{59047} = -2.61$<br>***p = 0.009***	$\beta = +0.00712$<br>$SE = 0.00423$<br>$t_{58317} = +1.68$<br>$p = 0.09$
HC (Below Factor 4 Median)	$\beta = -0.67$<br>$SE = 0.0835$<br>$t_{59047} = -8.03$<br>***p<0.0001***	$\beta = +0.00782$<br>$SE = 0.00197$<br>$t_{58317} = +3.96$<br>***p<0.0001***
PD	$\beta = -0.404$<br>$SE = 0.619$<br>$t_{59047} = -0.65$<br>$p = 0.51$	$\beta = -0.00989$<br>$SE = 0.0149$<br>$t_{58317} = -0.66$<br>$p = 0.51$
$adj - R^2$	0.86	0.59
N_{obs}	59059	58329
BIC	384867.83	-53787.05

Table S12: **Longitudinal changes in Reaction Time (RT) and grip strength from baseline (visit 1) to visit 3.** Models were specified as follows. Dependent Variable HandGripScores $\sim$ 1 + Age + Education + Gender + Age diff + Visit*group + (1 $|$Group) + (1 $|$SubjectID). Visit is a categorical variable defining baseline and visit 3. Control group (HC) was divided by median split based on Factor 4 scores, with HC above median as the base group against which other groups (PD, CVD and HC below median) are compared.
